# Planning for mARC treatments with the Eclipse treatment planning system

**DOI:** 10.1120/jacmp.v16i2.5351

**Published:** 2015-03-08

**Authors:** Vikren Sarkar, Long Huang, Prema Rassiah‐Szegedi, Hui Zhao, Jessica Huang, Martin Szegedi, Bill J. Salter

**Affiliations:** ^1^ Department of Radiation Oncology University of Utah Salt Lake City UT USA

**Keywords:** treatment planning, mARC, modulated arc

## Abstract

While modulated arc (mARC) capabilities have been available on Siemens linear accelerators for almost two years now, there was, until recently, only one treatment planning system capable of planning these treatments. The Eclipse treatment planning system now offers a module that can plan for mARC treatments. The purpose of this work was to test the module to determine whether it is capable of creating clinically acceptable plans. A total of 23 plans were created for various clinical sites and all plans delivered without anomaly. The average 3%/3 mm gamma pass rate for the plans was 98.0%, with a standard deviation of 1.7%. For a total of 14 plans, an equivalent static gantry IMRT plan was also created to compare delivery time. In all but two cases, the mARC plans delivered significantly faster than the static gantry plan. We have confirmed the successful creation of mARC plans that are deliverable with high fidelity on an ARTISTE linear accelerator, thus demonstrating the successful implementation of the Eclipse mARC module.

PACS numbers: 87.55.D‐, 87.55.ne, 87.57.uq,

## I. INTRODUCTION

Since 2012, Siemens has offered the possibility of delivering volumetric‐modulated arc (mARC) (Siemens AG, Munich, Germany) therapy on its family of linear accelerators. Multiple groups have published^1^, ^2^, ^3^, ^4^, ^5^, ^6^, ^7^, ^8^, ^9^, ^10^, ^11^ on the use of modulated arcs in the treatment of cancer in varying sites and they have shown that the technique is able to achieve dose distributions that are typically equivalent to those from static gantry IMRT plans, but that can be delivered with much higher efficiency. As already described in literature,^12^, ^13^, ^14^ Siemens' mARC uses the Burst Mode approach for treatment delivery, wherein segment dose is “burst” in at very high dose rates, over very short gantry angles. Because treatment planning systems typically approximate an arc delivery as a series of static beams, the Burst Mode approach should, theoretically, cause the delivered dose to more closely approach the calculated distribution from the treatment planning system.

Until recently, there was only one commercial planning system (Prowess Panther, Prowess Inc, Concord, CA) capable of planning for mARC deliveries. To address this limited availability of commercial mARC treatment planning system, a methodology^(15)^ was proposed to convert IMRT plans from any commercial treatment planning system into mARC plans. More recently, at least three other commercial systems have been released for planning for mARC treatments, namely 1) Monaco 5 (Elekta AB, Stockholm, Sweden), 2) RayStation 2.5 (RaySearch Laboratories AB, Stockholm, Sweden), and 3) Eclipse 13.5 (Varian Medical Systems, Palo Alto, CA). The purpose of this paper is to provide the first report on the clinical performance of the new Eclipse mARC module.

## II. MATERIALS AND METHODS

All of the test plans were created on a prototype Eclipse 13.5 system that was accessed through a citrix environment (Citrix Systems Inc., Fort Lauderdale, FL). A Siemens ARTISTE linac with a 160‐leaf MLC^16^, ^17^, ^18^ was modeled in the system using data collected as part of the commissioning of the linac. Models were created for the flattened 6 MV beam and unflat 7 MV.

All IMRT/mARC plans created for this project were optimized using version 13.5.07 of the photon optimizer (PO) algorithm. Per our clinical protocol, the final 3D dose distribution was calculated using the analytical anisotropic algorithm (AAA)^(19)^ (version 13.5.14). The dose grid was set to 2.5 mm with inhomogeneity corrections turned on.

As a first step to determine the capabilities of the planning system, the recommendations^(20)^ of Task Group 119 (TG‐119) of the American Association of Physicists in Medicine (AAPM) were used to create a set of plans. Since the TG‐119 results did not include any results of test plans run on an ARTISTE with the 160‐leaf MLC, both static gantry IMRT (SG‐IMRT) and mARC plans were created as part of this exercise.

The plans from TG‐119 recommendations were helpful to evaluate the general capabilities of both the planning and delivery systems. In order to evaluate a broader spectrum of clinically relevant treatment sites, plans were created for a total of 19 patients previously treated at our institution, with varying clinical treatment sites. Table 1 summarizes the types of plans that were created to verify the clinical relevance of the modality. The endpoints used for the planning process were either physician‐directed or as defined in RTOG protocols.

**Table 1:** Summary of plans developed for this project. SIB=simultaneous integrated boost, RSR=stereotactic radiosurgery, SBRT=stereotactic body radiation therapy

*Patient Number*	*Site*	*Prescription*	*Plan Modality*	*Planning End Points*	*Energy Used (MV)*
1	Kidney	2.4 Gy×15 fractions	mARC	Physician Guidelines	6
2	Abdomen	1.8 Gy×25 fractions	mARC	Physician Guidelines	6
3	Head & Neck	SIB : 2.25Gy/2 Gy/1.8Gy×30 fractions	mARC	Physician Guidelines	6
4	Head & Neck	SIB : 2.25Gy/2 Gy/1.8Gy×30 fractions	mARC	Physician Guidelines	6
5	Prostate	SBRT ‐ 7.25 Gy×6 fractions	mARC	Per RTOG 0938	7
6	Prostate	SBRT ‐ 7.25 Gy×6 fractions	mARC	Per RTOG 0938	7
7	Prostate	1.8 Gy×28 fractions followed by boost of 1.8 Gy×10 fractions	mARC	Physician Guidelines	7
8	Prostate	1.8 Gy×28 fractions followed by boost of 1.8 Gy×10 fractions	mARC	Physician Guidelines	7
9	Brain	2 Gy×23 fractions followed by boost of 2 Gy×7 fractions	mARC	Per RTOG 0825	7
10	Brain	4‐met SRS ‐ 15 Gy to each iso × 1 fraction (1 arc per met)	mARC	Physician Guidelines	7
		4‐met SRS ‐ 15 Gy to each iso × 1 fraction (single arc)	mARC	Physician Guidelines	7
11	Brain	SRS ‐ 20 Gy×1 fraction	mARC	Physician Guidelines	7
12	Brain	SRS ‐ 15 Gy×1 fraction	mARC	Physician Guidelines	7
13	Lung	SBRT ‐ 18 Gy×3 fractions	mARC	Physician Guidelines	7
14	Spine	SBRT ‐ 16 Gy×1 fraction	mARC	Per RTOG 0631	7
15	Spine	SBRT ‐ 16 Gy×1 fraction	mARC	Per RTOG 0631	7
16	Spine	SBRT ‐ 16 Gy×1 fraction	mARC	Per RTOG 0631	7
17	Spine	SBRT ‐ 18 Gy×1 fraction	mARC	Per RTOG 0631	7
18	Spine	SBRT ‐ 16 Gy×1 fraction	mARC	Per RTOG 0631	7
19	Spine	SBRT ‐ 16 Gy×1 fraction	mARC	Per RTOG 0631	7

All plans were sent to our record and verify (R&V) system (MOSAIQ version 2.41, IMPAC Medical Systems, Sunnyvale, CA) and delivered on our ARTISTE linear accelerator. The Delta^4^ device (Scandidos, Uppsala, Sweden) was used to perform dosimetric validation^(21)^ of all of the plans. Per our clinical protocol, the (3%, 3 mm) global gamma criterion was used to evaluate the results of the dose validation, with only points receiving doses higher than 10% of maximum evaluated, as suggested by TG‐119. The TG‐119 plans were also delivered on a Solid Water phantom (Gammex RMI, Middleton, WI) with an ion chamber to verify the dose to the chamber. The delivery time for each plan was measured in an attempt to characterize delivery efficiency of the mARC modality. As an added step to gauge efficiency, static‐gantry IMRT (SG‐IMRT) plans were created for a subset of the cases using the same end points as for the mARC plans. The delivery time of these SG‐IMRT plans were also measured for comparison to the corresponding mARC plan.

## III. RESULTS

**Figure 1:**
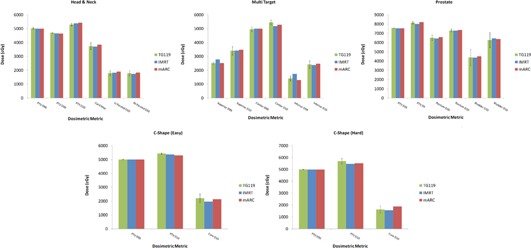
Summary of results from TG‐119 plans. The error bars show the standard deviation levels reported in the report.

The Eclipse module tested here was observed to be capable of creating treatment plans which met all of the planning criteria from TG‐119, except for the ‘Hard C‐Shape’, which the authors of the report admit to being likely unreachable. Even in the latter case, the metrics of concern were within 1 SD of the mean results reported by TG‐119. Figure 1 summarizes the relevant plan metrics from both the IMRT and mARC TG‐119 plans, while Table 2 shows the ion chamber results obtained for these plans.

**Figure 2:**
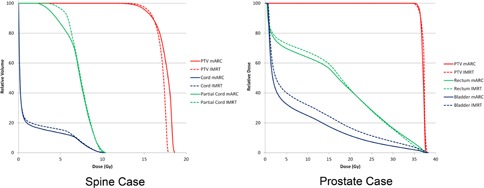
Example DVHs from corresponding mARC and IMRT plans for two of the clinical cases investigated. The spine case corresponds to case 19 from Table 1 and the prostate corresponds to case 5.

All of the 23 clinical mARC plans created were confirmed to be acceptable by meeting all of the planning criteria. For the 14 cases with a corresponding IMRT plan, similar coverage was obtained by the two plans. Figure 2 shows corresponding DVHs from two cases — a prostate case and a spine SBRT case.

Table 3 summarizes the results of the dose validations performed for the 33 mARC plans created for this study. The average gamma pass‐rate was 98.0%, with a standard deviation of 1.7%. The minimum pass‐rate was 94.2%, above our clinic's acceptance criterion of 90%.

Table 4 shows the delivery time comparison between the mARC plans and their corresponding SG‐IMRT counterparts. While the majority of mARC plans delivered in less time than the matching SG‐IMRT plan, with the latter taking up to three times as long to deliver, we note that there were two instances of prostate treatment sites where the SG‐IMRT plan delivered in less time than the mARC plan. A two‐tailed paired *t*‐test between the pairs of timing measurements gives a p‐value of 0.0003, showing statistically significant differences between the two sets.

**Table 2:** Ion chamber results from TG‐119 plans

*Case*	*Site*	*Dose per FX*	*Plan Type*	*Expected Dose (Gy)*	*Measured Dose (Gy)*	*Dose Difference (%)*
1	TG‐119 Easy C‐Shape	2	IMRT mARC	1.8 1.8	1.808 1.831	−1.7% 1.1%
2	TG‐119 Hard C‐Shape	2	IMRT mARC	1.9 1.9	1.848 1.919	−1.6% −0.2%
3	TG‐119 HN	2	IMRT mARC	1.9 1.9	1.929 1.885	−0.7% −0.6%
4	TG‐119 Multi‐Target	2	IMRT mARC	1.9 1.9	1.851 1.911	−0.9% 0.1%
5	TG‐119 Prostate	1.8	IMRT mARC	1.7 1.7	1.730 1.751	−0.4% 0.7%

**Table 3:** Results of the dose validations performed on the mARC plans developed

*Case*	*Site*	*Energy*	*Dose per FX (Gy)*	*Total Arcing Angle (°)*	*Total MUs mARC*	*Delivery Time (min)*	*Gamma (3%, 3 mm)*
1	TG‐119 Easy C‐Shape	6 MV	2	720	768.8	10.35	95.6
2	TG‐119 Hard C‐Shape	6 MV	2	720	778.5	10.30	96.4
3	TG‐119 HN	6 MV	2	360	584.0	5.83	98.8
4	TG‐119 Multitarget	6 MV	2	360	405.8	5.20	98.3
5	TG‐119 Prostate	6 MV	1.8	360	483.9	5.37	95.8
6	Kidney	6 MV	2.4	350	491	4.40	98.4
7	Abdomen	6 MV	1.8	360	864.7	10.77	99.3
8	Head & Neck	6 MV	2.25	720	699.2	10.32	98.5
9	Head & Neck	6 MV	2.25	720	517.7	9.83	99.1
10	Prostate	7 MV	7.25	720	2661.0	9.23	94.9
11	Prostate	7 MV	7.25	720	2751.5	9.37	95.1
12	Prostate	7 MV	1.8	360	1004.3	4.50	98.2
13	Prostate	7 MV	1.8	360	1241.0	4.53	94.2
14	Prostate	7 MV	1.8	360	481.9	4.33	99.5
15	Prostate	7 MV	1.8	360	1186.0	4.47	99.2
16	Brain	7 MV	2	360	596.6	4.25	95.8
17	Brain	7 MV	2	360	898.1	4.33	96.1
18	Brain	7 MV	15	1440	9235.5	23.03	100.0
19	Brain	7 MV	15	360	3233.8	5.53	99.6
20	Brain	7 MV	20	585	4484.7	8.65	98.6
21	Brain	7 MV	15	720	4287.2	9.77	98.5
22	Lung	7 MV	18	720	4646.7	9.50	100.0
23	Spine	7 MV	16	720	6748.8	10.73	97.5
24	Spine	7 MV	18	720	3817.1	11.23	99.7
25	Spine	7 MV	16	480	3911.0	8.30	97.9
26	Spine	7 MV	16	720	4672.8	11.82	98.2
27	Spine	7 MV	16	720	5192.4	10.18	99.0
28	Spine	7 MV	16	720	4447.2	8.25	99.0

**Table 4:** Comparison between delivery time for mARC plans and their corresponding SG‐IMRT plans

*Case*	*Site*	*Energy*	*Dose per FX*	*Delivery Rate (MU/min)*	*Total MUs*	*Delivery Time (min)*	*Total MUs*	*Delivery Time (min)*
1	TG‐119 Easy C‐Shape	6 MV	2	300	768.8	10.35	1372.5	13.68
2	TG‐119 Hard C‐Shape	6 MV	2	300	778.5	10.30	1410.7	13.28
3	TG‐119 HN	6 MV	2	300	584.0	5.83	1608.7	15.83
4	TG‐119 Multitarget	6 MV	2	300	405.8	5.20	512.4	7.48
5	TG‐119 Prostate	6 MV	1.8	300	483.9	5.37	344.9	6.07
6	Head & Neck	6 MV	2.25	300	699.2	10.32	1161.4	12.03
7	Head & Neck	6 MV	2.25	300	517.7	9.83	573.0	10.45
8	Prostate	7 MV	7.25	2000	2661.0	9.23	1664.2	8.08
9	Prostate	7 MV	7.25	2000	2751.5	9.37	3445.6	8.62
10	Prostate	7 MV	1.8	2000	1004.3	4.50	1215.1	13.02
11	Prostate	7 MV	1.8	2000	1241.0	4.53	1003.2	10.92
12	Prostate	7 MV	1.8	2000	481.9	4.33	1018.4	13.08
13	Prostate	7 MV	1.8	2000	1186.0	4.47	2032.6	10.40
14	Brain	7 MV	2	2000	596.6	4.25	613.8	7.88
15	Brain	7 MV	2	2000	898.1	4.33	550.7	7.82
16	Spine	7 MV	16	2000	5192.4	10.18	6039.4	13.73
17	Spine	7 MV	16	2000	4447.2	8.25	7704.6	14.92
18	Spine	7 MV	18	2000	3817.1	11.23	7974.6	27.25
19	Spine	7 MV	16	2000	3911.0	8.30	6545.8	24.50

## IV. DISCUSSION

As can be seen from Fig. 1, the TG‐119 IMRT plans created for the ARTISTE are comparable to the results reported by the Task Group from the multi‐institutional study. The figure also confirms that mARC plans can be obtained that are of similar quality to the IMRT plans. These results are comparable to those obtained by Mynampati et al.^(22)^ in their study using Eclipse with a Trilogy linear accelerator (Varian Medical Systems).

Our initial experience with the Eclipse mARC treatment planning module has shown that clinically relevant mARC treatment plans can be created for a wide array of sites. All of the 28 mARC plans (5 TG‐119 and 23 clinical) developed as part of this project were successfully delivered on the ARTISTE linear accelerator without delivery challenge or anomaly. The high gamma pass rate for the plans demonstrates that the machine and delivery approach were accurately modeled in the Eclipse system and that the developed treatment plans delivered with high fidelity. Although we used the AAA algorithm for our final dose calculation, we note that the tested Eclipse module also offers the more recently released Acuros^(23)^ calculation algorithm (Transpire Inc., Gig Harbor, WA) as a choice.

While the final dose calculation is faster for SG‐IMRT plans than for the mARC plans, the optimization step takes roughly the same time. Since the time for the final dose calculation is only a very small portion of the total planning time, our experience shows that the total planning time is comparable between mARC and SG‐IMRT plans, with planning time increasing for both with plan complexity. However, the delivery time comparison between the mARC plans and the corresponding SG‐IMRT plans makes clear the efficiency benefit of modulated arc plans.

## V. CONCLUSIONS

Preliminary tests performed on a new mARC‐capable calculation module for the Eclipse treatment planning system showed that clinically relevant plans could be efficiently created for multiple treatment sites. High gamma pass rates were observed for all delivered cases, demonstrating that the new module was capable of developing plans which deliver with high fidelity, and the generally shorter delivery times we observed for mARC compared to SG‐IMRT plans confirmed that the modality can be used to deliver high‐quality plans efficiently.

## ACKNOWLEDGEMENTS

The authors would like to express their sincere gratitude to Sergio Ibanez for the multiple hours he spent validating the plans developed as part of this project.
